# Exploration of pathways related to the decline in female circumcision in Egypt

**DOI:** 10.1186/1471-2458-13-921

**Published:** 2013-10-03

**Authors:** Sepideh Modrek, Jenny X Liu

**Affiliations:** 1 General Medical Disciplines, Stanford University School of Medicine, Palo Alto, CA, USA; 2 Global Health Sciences, University of California, San Francisco, CA, USA

**Keywords:** Female Circumcision, Adolescent health, Egypt

## Abstract

**Background:**

There has been a large decline in female genital circumcision (FGC) in Egypt in recent decades. Understanding how this change has occurred so rapidly has been an area of particular interest to policymakers and public health officials alike who seek to further discourage the practice elsewhere.

**Methods:**

We document the trends in this decline in the newest cohorts of young girls and explore the influences of three pathways—socioeconomic development, social media messages, and women’s empowerment—for explaining the observed trends. Using the 2005 and 2008 Egypt Demographic and Health Surveys, we estimate several logistic regression models to (1) examine individual and household determinants of circumcision, (2) assess the contributions of different pathways through which these changes may have occurred, and (3) assess the robustness of different pathways when unobserved community differences are taken into account.

**Results:**

Across all communities, socioeconomic status, social media messages, and women’s empowerment all have significant independent effects on the risk of circumcision. However, after accounting for unobserved differences across communities, only mother’s education and household wealth significantly predict circumcision outcomes. Additional analyses of maternal education suggest that increases in women’s education may be causally related to the reduction in FGC prevalence.

**Conclusions:**

Women’s empowerment and social media appear to be more important in explaining differences across communities; within communities, socioeconomic status is a key driver of girls’ circumcision risk. Further investigation of community-level women’s educational attainment for mothers suggests that investments made in female education a generation ago may have had echo effects on girls’ FGC risk a generation later.

## Background

Female genital circumcision (FGC), known alternatively as female genital mutilation and female genital cutting by the World Health Organization,^a^ involves the partial or complete removal of the external female genitalia. Although anthropologists have highlighted some differences in the significance of the practice across regions [1], FGC has been recognized as an entrenched cultural practice across the northern sub-Saharan region and along the Nile Valley, where it is thought to have originated. There, FGC is most often described as a rite of passage for young girls a way to protect daughters' modesty and improve their marriage prospects—and is mainly transmitted across generations through women [2,3]. In Egypt, the practice was nearly universal until recently [4,5] and is typically performed on girls between the ages of eight and fourteen, preferably before the onset of puberty [6,7].

Notably, there has been a large decline in female circumcision in Egypt in recent decades [5] and an increase in its “medicalization” in which the circumcision is performed by a health professional [6-8]. Laws passed in 1959 and 1978 prohibiting female circumcision without a clear medical indication went largely unenforced [3,4] and concerted efforts to discourage FGC reemerged in the mid-1990s. In particular, FGC was a key topic at the 1994 United Nation’s International Conference on Population and Development (ICPD) in Cairo, sparking national debate and action from civil groups to “eradicate” female circumcision through education, research, and advocacy [4,9]. Subsequently, FGC was officially banned in 1997, but the loophole allowing for medically necessary circumcision was only eliminated in 2007 following outrage over the death of an 11-year old girl after being circumcised [10,11]. Non-governmental organizations (NGOs) have been the most active in raising awareness and promoting anti-circumcision messages, which are now included in most community development, health care, and women’s right programs in Egypt [12].

Understanding how FGC has declined internationally and in Egypt in particular is an area of active research [13]. Internationally, research has focused on studying the myriad of strategies that have been used to increase knowledge on the health risks associated with circumcision through social media messages, conversion of circumcision practitioners’ beliefs, public statements against the practice, and the imposition of laws banning circumcision [14]. Egypt is of particular interest because the declines in circumcision rates have been rapid. According to Egypt Demographic and Health Survey (EDHS) estimates, fewer than 40 percent of girls born in the mid-1990s are circumcised by age 13 compared to nearly 90 percent of girls born in the 1980s. Likewise according to the Survey of Youth in Egypt (SYPE), 52 percent of girls aged 10–14 were circumcised compared to 90 percent of girls aged 25–29 in 2009 [7]. The impact that the national public debate and social marketing initiatives against FGC have had on accelerating this decline is an area of interest to policymakers and public health officials alike.

Previous efforts to elucidate the factors that have contributed to the decline in FGC in Egypt, using a nationally representative sample, have focused on anti-FGC health information campaigns sparked by the 1994 ICPD. While there is some evidence that FGC-specific social media messages have influenced changes in popular attitudes [15], the evidence linking changes in girls’ circumcision risk to the 1994 ICPD event itself is only suggestive [4]. Other facilitators of cultural change that may also have contributed and accelerated the abandonment of FGC among Egyptian families have yet to be explored despite similarly large changes over recent decades. Most notably, general socioeconomic status (SES) has improved as the country has developed, women’s education has increased even more as a result of concerted government programs [16], and the predominately young population is increasingly exposed to Western cultural influences. Based on 1995 representative sample of ever-married women (aged 17–55 years) in Minya, Egypt, Yount found that mother’s education, though not father’s, was negatively associated with daughter’s circumcision status [17]. Tag-Eldin and colleagues also provide descriptive evidence from a nationally representative sample of schoolgirls that development and modernization may also be important drivers of declining FGC [5].

To build on these findings and more broadly explore different pathways through which changes in FGC have taken root in Egypt, we collate data from the 2005 and 2008 EDHSs—the most recent population-wide data available on the prevalence of FGC in Egypt—to achieve two aims. First, we update the trend in FGC among the newest cohorts of girls at risk. Second, through a series of logistic regression models, we assess the relative contributions of different determinants of the continued circumcision of young girls: (1) general economic development, and particularly maternal education, (2) information on FGC through social media exposure, (3) women’s empowerment, and (4) community norms. Each of these factors may have independently and/or jointly facilitated the rapid decline of FGC in Egypt. Lastly, we further investigate the role of maternal education in predicting circumcision risk since mothers are the primary decision-maker for FGC and because Egyptian women have also experienced an unprecedented rise in educational attainment since the 1960s. We conduct a series of supplementary analyses to assess whether changes in educational opportunities for the generation of mothers may be causally related to the changes in circumcision risk for today’s generation of daughters.

## Methods

### Data

Five waves of the Egypt Demographic and Health Surveys (EDHSs), which are publicly available at http://www.measuredhs.com, are collated to create two datasets. The first dataset organizes the daughter-level circumcision outcomes by combining the 2005 and 2008 EDHSs. For a subset of daughters, we examine the role of maternal education in more detail and add additional information on area-level women’s educational attainment. The smaller, second dataset uses all EDHSs (1992, 1995, 2000, 2005, and 2008) to construct area- and cohort-specific measures of women’s educational attainment and is linked to the sample of daughters based on the mother’s year of birth and area where she would have experienced her primary education. Each dataset is described below.

#### Primary dataset of daughters and their circumcision status

The main analyses of girls’ risk of circumcision uses data from the 2005 and 2008 EDHSs. In both waves, ever-married women aged 15–49 were asked about their experiences with female genital circumcision, their attitudes toward the practice, the FGC status for each of their daughters under 17 and 19 years old in 2005 and 2008, respectively. Earlier EDHS waves are not used because circumcision questions changed between survey waves and are incompatible with the later and more complete sets of circumcision-related questions. Daughters’ circumcision status is the main outcome of interest, defined as an indicator variable. Because younger girls are unlikely to be at risk of circumcision, the sample is restricted to girls aged 8–18 (born 1989–2000 at the time of survey)^b^. The final dataset of daughters at risk for circumcision has 17,579 observations. These data were collected from households embedded in 2,551 primary sampling units or clusters. In the first set of analyses, each cluster is treated as a community.

#### Sub-sample of daughters with additional area-level variables for women’s educational attainment

For supplementary analyses of maternal education, we add area-level variables for women’s educational attainment linked to mother’s birth year and area of residence during her childhood. Educational attainment for ever-married women is asked in all waves of the EDHS and can be pooled to calculate the average educational attainment of all women within a given area by birth cohort. To create consistent geographic area units across EDHS waves, GIS coordinates of the sampled clusters in each EDHS wave are mapped to sub-governorate area-level units only available in the 1992 EDHS. For each strata sampled in the 1992 EDHS, we map the GIS coordinates of the center of that strata and capture the 30 closest clusters to this centroid in subsequent surveys. To exclude more distant communities that may be less similar to others in the area, clusters more than 20 kilometers (12.4 miles) from the centroid in urban areas or more than 30 kilometers (18 miles) away in rural areas are dropped. While not strictly based on administrative distinctions or precise neighborhoods, these GIS-based areas should represent a cohesive geographic area and are only used in the supplemental analysis. A detailed explanation of the matching procedure to create sub-governorate geographic units is given in Additional file 1. Pooling across clusters and survey waves allows for a more precise calculation of women's education for each birth cohort, spanning 25 years (1956–1980) within each geographic sub-governorate area. A five-year moving average is then applied to smooth trends across individual years for each of the following proportions: less than primary school; completed primary school, but not secondary; and completed secondary or more.

Area-level women’s educational attainment can then be linked to mothers in the primary dataset of daughters by the mother’s birth year and area of residence. A total of 7,696 observations are lost during the linking process for two reasons. First, some clusters randomly sampled in later EDHS waves cannot be assigned to pre-defined sub-governorate units from the 1992 wave (see Additional file 1 for further details). This matching process accounts for the majority of daughter observations lost. Importantly, because Frontier governorates were not sampled in earlier EDHS waves, they are essentially excluded in the sub-sample. Second, only mothers who report residing in the same community since age 6 (the age for entering primary school) at the time of survey are included; area-level women’s educational attainment cannot be linked for women who have moved. Measures of the area-level women’s educational attainment are more likely to reflect the aggregate result of women’s educational opportunities during the time that mothers were growing up.

### Data analysis

#### Descriptive analysis

To update the historical trend in FGC with the most recent change, data from the 2008 EDHS are combined with previous waves (1995, 2000, 2003 interim, 2005, and 2008). The proportion of all daughters who are either circumcised, or are likely to be circumcised in the future because of mothers’ intentions, can be calculated and plotted by birth cohort. Proportions are not corrected for population sample weights as the data are not intended to be representative of a particular cross-sectional year, but rather an aggregate of individual observations across cohorts.

#### Pathway analysis

A series of logistic regression models are estimated to assess the contributions of different individual, household, and environmental factors for the risk of circumcision among young girls. Generally, the likelihood that a girl is circumcised can be specified as:

$$\mathrm{Pr}\left(Y_{\mathrm{ic}}=1\right)=\Gamma \left(X_{\mathrm{ic}},M_{\mathrm{ic}},H_{\mathrm{ic}},N_{c}\right)$$

where *Y*_*ic*_ represents the circumcision status for girl *i* in area *c*; *X*_*ic*_ includes controls for individual characteristics; *M*_*ic*_ represents her mother’s characteristics; *H*_*ic*_ stands for other household characteristics; and *N*_*c*_ represent the community in which her family is embedded. To account for community effects, *N*_*c*_, we use two types of models: (1) a community random effects model that accounts for correlation of circumcision risk within communities but does not isolate within-community differences, and (2) a community fixed-effects model to isolate within-community differences and control for unobserved differences across communities. In addition, because we include two survey waves collected in different years, all models include a series of interaction terms for survey year and daughters’ year of birth to account for secular trends and right censoring in our data (i.e. although 96 percent are circumcised by age 13, the sample is restricted to those under age 17 in 2005 and 19 in 2008, and the entire period for which girls are at risk is not fully observed).

Our base model includes a minimum set of explanatory variables, including individual (daughter’s birth year and birth order), maternal (year of birth, circumcision status, age at first marriage, number of children ever born, religion), and household-level (household size, governorate and urban/rural) demographic characteristics. A variety of other variables are included as indicators for different hypothesized pathways: (1) Indicators for economic development and modernization include SES measures, such as mother’s education level, mother's labor force participation, father’s education level, and household wealth quintiles. (2) Exposure to information on FGC through FGC-specific social media (i.e. targeted toward beliefs about the practice) can be measured with an indicator based on whether mothers report hearing about or discussed FGC issues through any social medium (i.e. television, magazine, radio, community meetings, leaders, discussions with friends or family). (3) Even if women do not support FGC, they may opt to continue the practice if they do not feel empowered to make a decision that deviates from expected norms. To explore the role of women’s empowerment, we use two measures of empowerment: (a) a principal components score of four survey questions that ask about having control over certain types of household decisions (i.e. allocation of household resources, decisions regarding her own health, and being able to visit others); and (b) a second principal components score based on four questions measuring tolerance towards domestic violence (i.e. is domestic violence okay if a woman neglects child care duties, goes out without permission, withholds sex, or argues?). (4) Finally, we control for unobserved community norms at different levels of geographic size through stratification of the regression models by community to only compare daughters within the same community to each other. Although we recognize that a simple geography-based measure of community is unlikely to embody the socio-cultural context in which households are residing, this is the closest proxy available in the existing data to effectively control for unobservable community-level effects.

#### Supplemental maternal education analysis

An additional series of regressions are estimated to further examine the robustness of the role of maternal education and account for other confounders in estimating a causal effect of maternal education on circumcision risk. Because education is non-randomly assigned, some women will obtain more schooling than others based on unobserved ability or parental preferences. We first rerun the preferred logistic regression model including cluster-level fixed effects on the subsample of daughters to assess any differences in estimates purely due to differences in sample composition. We then substitute larger, constructed sub-governorate area-level units for smaller community cluster units to minimize the number of observations that are dropped due to having no within-cluster variation in circumcision outcomes^c^. While the units used for fixed effects represent larger geographic areas, confounding of the estimated coefficients on maternal education variables due to unobserved differences across units can be similarly controlled for. Further, unobserved differences in communities’ trajectories of development can additionally bias the effect of mothers’ education on daughters’ circumcision. For example, cultural attitudes toward women may change at different paces across communities, affecting both preferences for women’s education and circumcision. As such, we include a vector of area-level dummies interacted with mother’s year of birth to account for these community-specific unobserved trends and test for residual confounding.

Finally we present a ‘reduced form’ regression where individual mother’s education is replaced with plausibly exogenous variation in area- and cohort-specific women’s educational attainment. Younger cohorts of women have progressively experienced greater educational opportunities over time in Egypt as a result of concerted policies aimed at building more schools and making primary schooling compulsory beginning in the 1960s [18]. This historical rise in women’s educational opportunities suggests that women’s educational attainment a generation ago, resulting from a myriad education policies, may be exogenous and unrelated to daughter’s circumcision risk a generation later. This final analysis reflects the overall effect of changing women’s education on circumcision, through changes in both individual realized education itself and area-level norms.

#### Ethical considerations

In accordance with federal regulations, this work has been deemed to be exempt from full review by the Committee for the Protection of Human Subjects at Stanford University because it uses de-identified publically available data.

## Results

### Descriptive analysis

**Figure 1:**
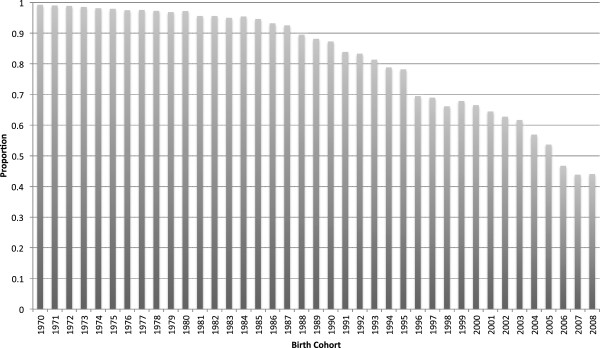
**Proportion of girls who are circumcised or whose mothers intend to have them circumcised, by birth cohort.** NOTE.—Authors’ calculations based on compiling 1995, 2000, 2003 interim, 2005 & 2008 Egypt Demographic and Health Surveys.

Updated estimates of the proportion of girls who are circumcised by birth cohort from 1970 to 2008 are shown in Figure 1. For more recent cohorts born in 2000, the proportion circumcised also includes girls whose mothers report an intention to circumcise them, but were not yet circumcised at the time of survey. Before 1987, over 90 percent of girls are circumcised. However, the percentage steadily declines thereafter. For cohorts born since 2005, fewer than 50 percent of girls are expected to be circumcised.

### Sample characteristics

Summary statistics for both the main analytic sample of daughters (column 1) and the sample subset with additional maternal variables (column 2) are provided in Table 1. Overall, the main analytic sample of 17,579 daughters (linked to 11,695 mothers) is quite similar to the subsample. Girls are 12.7 years old on average. Of the 50 percent of girls who are circumcised, the average age at circumcision is 9.3 years. In contrast, 96 to 98 percent of mothers are circumcised. The average mother first marries at age 18.7 and her current spouse is about 12 years older. About 23 percent of mothers work. With regard to education, about 57 percent of mothers have less than primary schooling, 13 percent have completed primary school but not secondary school, and about 30 percent have completed secondary school or more. Comparatively, only 44 percent of fathers have less than primary school education, 16 percent have completed primary, and about 40 percent have completed secondary or higher education. Over 75 percent of mothers have been exposed to at least one type of media message about female circumcision. The empowerment score for household decision-making ranges from −3.5 to 1.4 (SD = 1.4) and the range for the empowerment index based on domestic violence questions is from −1.8 to 3.0 (SD = 1.7). The main difference in the samples has to do with geographic location for mothers in the subsample: in the main sample, 39 percent of daughters live in urban areas and 6 percent are from Frontier governorates, but only 32 percent of observations in the restricted sample are in urban areas and all girls from Frontier governorates are excluded.

**Table 1:** Sample characteristics

	**(1) Analytical sample (N = 17,579)**		**(2) Sub-sample linked to mother’s childhood location (N = 9,883)**	
	**Mean**	**SD**	**Mean**	**SD**
***Daughter characteristics***				
Age	12.65	3.04	12.69	3.04
Circumcised	0.49	0.50	0.51	0.50
Age at circumcision if circumcised	9.25	7.51	9.30	7.63
First born	0.24	0.43	0.24	0.43
Second born	0.23	0.42	0.23	0.42
Third born	0.19	0.39	0.18	0.39
Fourth born & higher	0.33	0.47	0.35	0.48
***Mother characteristics***				
Year of birth	1968	5.57	1968	5.58
Circumcised	0.96	0.19	0.98	0.14
Age at first marriage	18.67	3.93	18.50	3.87
Age difference with husband	12.45	7.18	12.39	7.07
Number of Children	4.95	2.10	5.02	2.12
Muslim	0.95	0.22	0.95	0.21
***Household characteristics***				
Household size: 2 to 4	0.08	0.27	0.07	0.26
Household size: 5 to 7	0.62	0.49	0.62	0.49
Household size: 8 to 10	0.22	0.41	0.22	0.42
Household size: 11+	0.09	0.29	0.09	0.29
***Geographic location***				
Urban	0.39	0.49	0.32	0.47
Urban governorates	0.15	0.35	0.12	0.32
Lower Egypt urban	0.09	0.28	0.09	0.28
Lower Egypt rural	0.23	0.42	0.27	0.45
Upper Egypt urban	0.13	0.33	0.11	0.32
Upper Egypt rural	0.35	0.48	0.41	0.49
Frontier governorates	0.06	0.23	0.00	
***Socioeconomic Status***				
Wealth quintile 1	0.26	0.44	0.28	0.45
Wealth quintile 2	0.20	0.40	0.22	0.42
Wealth quintile 3	0.19	0.39	0.19	0.39
Wealth quintile 4	0.17	0.38	0.16	0.36
Wealth quintile 5	0.18	0.38	0.15	0.36
Mother works	0.23	0.42	0.23	0.42
Mother less than primary	0.57	0.50	0.59	0.49
Mother completed primary	0.13	0.33	0.12	0.33
Mother completed secondary or more	0.30	0.46	0.29	0.45
Father less than primary	0.44	0.50	0.47	0.50
Father completed primary	0.16	0.37	0.16	0.37
Father completed secondary or more	0.40	0.49	0.37	0.48
***Anti-FGC Messaging***				
Exposed to 1 or more messages	0.77	0.42	0.76	0.43
***Empowerment Indexes***				
PCA of household decisions [Rng −3.5–1.3]	0.06	1.44	0.08	1.42
PCA of Anti-domestic violence [Rng −1.8–3.0]	0.05	1.76	0.09	1.77
Observations from 2005 Wave	9784		4503	
Observations from 2008 Wave	7795		5380	

### Pathway analysis using analytical sample

Table 2 displays the odds ratios and 95 percent confidence intervals for logistic regression estimates of the likelihood of circumcision for girls born between 1989 and 2000. Column 1 presents results for the base model with cluster-level random effects and can be compared to estimates in columns 2 to 4 which present results for each of three sets of pathway variables that are incrementally added: SES, exposure to FGC messaging, and empowerment, respectively. Lastly, estimates in column 5 are the result of adding cluster-level fixed-effects, which essentially stratifies the analysis by cluster and compares only girls within clusters to each other.

**Table 2:** Pathway analysis of the association between daughters’ circumcision status and several hypothesized variables

	**Daughter circumcised**				
	**(1) Base model**	**(2) Add SES**	**(3) Add FGC messaging exposure**	**(4) Add empowerment**	**(5) Add cluster FE**
***Mother characteristics***					
Year of birth	0.997	0.997	0.997	0.997	1.001
	(0.980–1.015)	(0.979–1.014)	(0.980–1.014)	(0.980–1.015)	(0.982–1.020)
Circumcised	63.388**	54.850**	57.473**	57.382**	16.206**
	(33.478–120.021)	(28.868–104.219)	(30.166–109.501)	(30.103–109.380)	(8.295–31.662)
Age at first marriage	0.937**	0.969**	0.969**	0.970**	0.963**
	(0.918–0.956)	(0.948–0.990)	(0.949–0.990)	(0.949–0.991)	(0.940–0.986)
Age difference with husband	0.985*	0.987*	0.988*	0.987*	0.986*
	(0.974–0.997)	(0.976–0.999)	(0.976–0.999)	(0.976–0.999)	(0.973–0.998)
Number of Children	1.018	1.006	1.005	1.003	0.999
	(0.974–1.063)	(0.963–1.051)	(0.962–1.050)	(0.960–1.048)	(0.953–1.048)
Muslim	5.344**	5.416**	5.422**	5.371**	6.044**
	(4.022–7.099)	(4.071–7.206)	(4.075–7.215)	(4.037–7.145)	(4.361–8.379)
***Daughter characteristics***					
*Birth order* (*relative to 4th or higher*)					
First born	0.824	0.885	0.880	0.874	0.903
	(0.656–1.036)	(0.703–1.115)	(0.699–1.107)	(0.694–1.101)	(0.704–1.157)
Second born	0.946	1.012	1.008	1.007	1.049
	(0.776–1.153)	(0.829–1.236)	(0.826–1.231)	(0.825–1.229)	(0.846–1.301)
Third born	0.914	0.952	0.952	0.950	0.972
	(0.770–1.086)	(0.800–1.132)	(0.800–1.132)	(0.799–1.131)	(0.807–1.170)
***Household characteristics***					
*Household size* (*relative to Household size*: *2 to 4*)					
Household size: 5 to 7	0.943	0.995	0.998	1.000	1.127
	(0.700–1.269)	(0.738–1.342)	(0.740–1.346)	(0.741–1.350)	(0.813–1.563)
Household size: 8 to 10	1.008	1.080	1.082	1.087	1.106
	(0.819–1.239)	(0.877–1.329)	(0.879–1.331)	(0.882–1.338)	(0.880–1.389)
Household size: 11+	1.089	1.114	1.112	1.116	1.112
	(0.878–1.350)	(0.898–1.382)	(0.896–1.379)	(0.899–1.385)	(0.878–1.408)
***SES***					
Wealth quintile 1		0.728**	0.719**	0.705**	0.779*
		(0.607–0.873)	(0.599–0.863)	(0.587–0.847)	(0.636–0.953)
Wealth quintile 2		1.023	1.023	1.015	1.000
		(0.861–1.216)	(0.861–1.216)	(0.853–1.206)	(0.827–1.210)
Wealth quintile 4		0.692**	0.695**	0.701**	0.758**
		(0.575–0.834)	(0.577–0.837)	(0.582–0.844)	(0.619–0.930)
Wealth quintile 5		0.449**	0.450**	0.457**	0.548**
		(0.359–0.562)	(0.360–0.563)	(0.365–0.572)	(0.423–0.709)
Mother works		0.662**	0.663**	0.662**	0.735**
		(0.579–0.757)	(0.579–0.758)	(0.578–0.757)	(0.636–0.850)
Mother completed primary		0.836*	0.842	0.854	0.692**
		(0.700–0.999)	(0.705–1.005)	(0.715–1.021)	(0.568–0.842)
Mother completed secondary or more		0.546**	0.552**	0.567**	0.591**
		(0.452–0.660)	(0.456–0.667)	(0.468–0.687)	(0.480–0.728)
Father completed primary		1.072	1.077	1.077	1.018
		(0.915–1.256)	(0.919–1.262)	(0.919–1.261)	(0.858–1.208)
Father completed secondary or more		1.111	1.121	1.132	1.038
		(0.945–1.307)	(0.953–1.318)	(0.962–1.331)	(0.868–1.242)
***Anti-FGC Messaging***					
Exposed to 1 or more messages			0.828**	0.838**	0.872
			(0.725–0.944)	(0.734–0.957)	(0.753–1.010)
***Empowerment Indexes***					
PCA of household decisions				0.999	1.014
				(0.959–1.040)	(0.970–1.060)
PCA of tolerance of domestic violence				1.042*	1.028
				(1.006–1.079)	(0.989–1.067)
Daughter YOB & Wave FE	YES	YES	YES	YES	YES
Urban FE	YES	YES	YES	YES	NO
Region FE (5 units)	YES	YES	YES	YES	NO
Cluster RE	YES	YES	YES	YES	NO
Cluster FE	NO	NO	NO	NO	YES
Observations	17579	17579	17579	17579	13755
Number of Clusters	2551	2551	2551	2551	1714

Note: Odds ratios and 95% confidence intervals presented. ** p < 0.01, * p < 0.05.

Across all model specifications, mother’s characteristics are consistently associated with daughters’ likelihood of circumcision. In the base model, mother’s circumcision status and religion (i.e. being Muslim) are the strongest predictors. There are also statistically significant associations with mother’s age at first marriage and her age difference with her husband, though the magnitudes of the parameter estimates are comparatively smaller. When SES variables are added (column 2), mothers who work and those who have at least completed primary school are significantly less likely to circumcise their daughters by one-third to nearly half. In addition, household wealth exhibits a curvilinear relationship with circumcision. Households in the bottom or the top two wealth quintiles are less likely to circumcise their daughters compared to the middle quintiles. SES variables are consistently significant even after adding measures of FGC message exposure and empowerment. Exposure to at least one FGC message is also negatively and independently associated with the likelihood of circumcision (column 3). When empowerment indexes are added (column 4), only the scale measuring tolerance for domestic violence is associated with daughter’s FGC status, suggesting that mothers who are more tolerant of domestic violence are also more likely to circumcise their daughters.

In the specification presented in column 5, the regression is stratified by community and a fixed effect is added for each sample cluster. Due to a lack of within-community variation in circumcision status among girls (i.e. either 100 or 0 percent of girls in the community were circumcised), 3,824 observations from 834 community clusters are dropped. When time-invariant unobserved differences across communities are controlled for, only a few predictors remain significant and some estimated coefficients change substantially. This suggests that some unobservable heterogeneity across communities is correlated with both girls’ circumcision risk and many individual predictors. Most notably, the estimated coefficient on mother’s circumcision status reduces 3.5 fold from an odds ratio of 57.4 to 16.2. Other independent variables that remain significant with cluster fixed effects include mother’s age at first marriage, her education, and her labor force participation. In particular, when only looking within communities, the magnitude and significance of the association between mother’s who have completed primary school and daughter’s circumcision status becomes much stronger (when stratified by cluster with fixed effects: OR = 0.69, 95 percent CI 0.57-0.84; when stratified by region with cluster random effects: OR = 0.85, 95 percent CI 0.72-1.02); the point estimate and significance for mother’s secondary schooling or more is unchanged. While the cluster fixed effects specification shows substantively similar results for socioeconomic indicators with somewhat smaller estimated effects, results for media exposure and empowerment indices are no longer statistically significant. Father’s education is not significant in any model specification. Hausman tests performed to assess the differences in estimates between random and fixed effect specifications indicate that the inclusion of fixed effects is necessary to make the parameter estimates more consistent (p < 0.01). Therefore, the specification in column 5 with fixed-effects is the preferred specification.

### Supplemental analyses exploring maternal education

**Figure 2:**
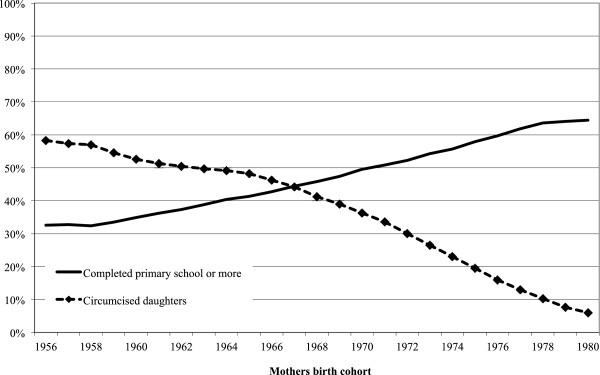
**Female circumcision among daughters and women’s education by mothers’ birth cohort, 1956–1985.** SOURCE.—1992, 1995, 2000, 2003 interim, 2005, and 2008 Egypt Demographic and Health Surveys. NOTE.—Lines are smoothed using a five-year moving average.

Although maternal education becomes a stronger predictor of daughter’s circumcision status within communities, it is unclear whether education itself is related to the continuance of female circumcision or whether other factors yet unaccounted for—individual preferences, gender norms, general economic development—may be driving both trends. Descriptively, there is a striking and inverse relationship between increases maternal education for mothers and the FGC status of their daughters (see Figure 2): as education rises for cohorts of mothers over time, the proportion of their daughters who are circumcised plummets. However, this simple relationship does not account for a variety of other confounders that may explain both trends.

Table 3 presents a series of analyses for the sub-sample of women for whom we can link in an additional area-level variable for the historical upward trend in women’s education. As a comparison, column 1 displays results that include cluster-level fixed effects to assess any differences in estimates purely due to the composition of the sub-sample. Overall, estimates are similar with few exceptions. The largest difference is the increase in the magnitude of the effect of Muslim religion (from OR = 6.0 to OR = 9.0). Point estimates for higher wealth quintiles are slight larger, while those of female labor force participation and education are essentially the same.

**Table 3:** Supplemental analysis of association between daughters’ circumcision status and maternal education

	**Daughter circumcised**			
	**Restricted sample with mother’s childhood location (N = 9883)**			
	**(1) Cluster FE**	**(2) Area FE**	**(3) Area trends**	**(4) Reduced form**
***Mother characteristics***				
Year of birth	0.989	1.008	0.888**	1.025*
	(0.963–1.016)	(0.988–1.028)	(0.860–0.917)	(1.000–1.050)
Circumcised	14.703**	9.695**	9.849**	10.098**
	(5.332–40.543)	(4.786–19.639)	(3.742–25.918)	(4.975–20.494)
Age at first marriage	0.95882*	0.984	0.986	0.976
	(0.927–0.992)	(0.960–1.009)	(0.956–1.017)	(0.953–1.000)
Age difference with husband	0.981	0.981**	0.979*	0.980**
	(0.962–1.001)	(0.967–0.995)	(0.962–0.997)	(0.967–0.994)
Number of Children	0.975	0.987	0.977	0.983
	(0.911–1.043)	(0.940–1.036)	(0.918–1.040)	(0.935–1.032)
Muslim	9.004**	7.340**	7.295**	7.338**
	(5.450–14.875)	(5.409–9.960)	(4.567–11.653)	(5.403–9.966)
***Daughter characteristics***				
*Birth order* (*relative to 4th or higher*)				
First born	1.099	0.807	0.791	0.781
	(0.774–1.560)	(0.619–1.053)	(0.609–1.026)	(0.599–1.020)
Second born	1.104	0.919	0.911	0.892
	(0.816–1.495)	(0.729–1.158)	(0.719–1.153)	(0.707–1.124)
Third born	1.188	1.012	1.008	0.996
	(0.909–1.552)	(0.825–1.242)	(0.817–1.243)	(0.811–1.222)
***Household characteristics***				
*Household size* (*relative to Household size: 2 to 4*)				
Household size: 5 to 7	1.121	0.935	0.874	0.900
	(0.699–1.796)	(0.667–1.311)	(0.570–1.341)	(0.642–1.264)
Household size: 8 to 10	1.172	0.986	0.977	0.951
	(0.859–1.598)	(0.789–1.232)	(0.732–1.304)	(0.761–1.190)
Household size: 11+	1.356	1.096	1.098	1.072
	(0.984–1.870)	(0.868–1.385)	(0.835–1.443)	(0.847–1.356)
***SES***				
Wealth quintile 1	0.826	0.862	0.836	0.906
	(0.627–1.088)	(0.710–1.047)	(0.641–1.092)	(0.747–1.099)
Wealth quintile 2	0.899	1.000	1.001	1.031
	(0.693–1.165)	(0.829–1.206)	(0.795–1.260)	(0.855–1.243)
Wealth quintile 4	0.644**	0.696**	0.691**	0.657**
	(0.478–0.868)	(0.564–0.860)	(0.539–0.886)	(0.533–0.809)
Wealth quintile 5	0.509**	0.445**	0.428**	0.404**
	(0.344–0.754)	(0.346–0.572)	(0.320–0.574)	(0.316–0.517)
Mother works	0.782*	0.807**	0.811*	0.776**
	(0.638–0.960)	(0.696–0.934)	(0.675–0.974)	(0.671–0.897)
Mother completed primary	0.629**	0.757**	0.770*	
	(0.470–0.842)	(0.617–0.929)	(0.597–0.994)	
Mother completed secondary or more	0.611**	0.622**	0.614**	
	(0.450–0.830)	(0.499–0.776)	(0.466–0.808)	
Father completed primary	1.185	1.175	1.206	1.127
	(0.926–1.516)	(0.983–1.405)	(0.967–1.504)	(0.944–1.345)
Father completed secondary or more	1.055	0.949	0.932	0.778**
	(0.814–1.368)	(0.791–1.138)	(0.750–1.159)	(0.663–0.912)
***Area women’s educational attainment***				
Proportion of women completing primary				0.226**
				(0.076–0.674)
Proportion of women completing secondary or more				0.441
				(0.192–1.010)
Daughter YOB & Wave FE	YES	YES	YES	YES
Cluster FE	YES	NO	NO	NO
Area FE	NO	YES	YES	YES
Area* time trend FE	NO	NO	YES	NO
Observations	7443	9806	9806	9739
Number of clusters	1089			
Number of Areas		167	167	167
Observations dropped because no variation in outcome within geographic unit	2440	77	77	144

Note: Odds ratios and 95% confidence intervals presented. ** p < 0.01, * p < 0.05.

Because large numbers of observations must be dropped when community-level cluster fixed effects are added, a greater number of observations can be retained when larger sub-governorate geographic units are substituted for smaller cluster designations. This larger area unit is also more likely to reflect the results of historical educational opportunities for women than the primary sampling cluster units within the EDHSs. Since assessing the area-level association of rising women’s education is the main goal of this sub-analysis, larger sub-governorate area units are used in specifications presented in columns 2–4. Column 2 displays results that control for sub-governorate area fixed effects (rather than cluster-level fixed effects). The sample size increases to 9,806 observations for 167 sub-governorate units. While the estimated associations for mother’s circumcision status and mother’s religion on daughters’ FGC risk are somewhat a smaller, all other estimated coefficients remain substantively unchanged. In particular, the estimated odds ratios for maternal education remains similar across the two models and similar to those estimated for the larger sample of daughters, suggesting that this association is robust even when the most stringent methods are applied to control for possible confounding in this relationship. This remains the case even when area-specific time trends are added to the model (column 3), indicating that there is little residual confounding from unobserved time trends that may be unique to each area. Only the odds ratio for mother’s year of birth becomes statistically significant—mother born to later cohorts are less likely to circumcise their daughters. Finally, when individual mothers’ realized education is replaced with the average attainment level of her cohort peers in the area where she went to school (i.e. the reduced form in column 4), the proportion of women who complete primary school remains statistically significant. The estimated coefficient indicates that a 10 percent increase in the proportion of women peers who complete primary school in the area is related to approximately a 3.5 percentage point decrease in the proportion of daughters who are circumcised a generation later. The estimated odds ratio for the proportion of women who complete secondary school or more is marginally significant (p-value < 0.053), suggesting that a 10 percent increase in the proportion of women/peers who complete secondary school or more is associated with a two percentage point decrease in the proportion of daughters who are circumcised a generation later.

The reduced form specification also allows us to look at the effect of father’s education independently of the sorting and selection into marriage. Since women with higher education tend to marry men with higher education, the correlation coefficient between mothers with more than secondary education and fathers with more than secondary education is 0.72 (p-value < 0.01). While this correlation is significant, supplemental analysis (not shown) asserts that the sample size is sufficient so that multicollinearity in the parameter estimates does not inhibit a consistent estimate of the independent effects.^d^ In the reduced form specification, fathers with more than secondary education are less likely to circumcise their daughters, but the magnitude of the coefficient is much smaller than the estimates for mother’s education. In addition, mother’s education shows a graded relationship—the more education a mother has, the less likely she will be to circumcise her daughter—unlike fathers’ education which does not show a similar pattern.

## Discussion

Our analysis of data from the 2005 and 2008 waves of the Egypt Demographic and Health Surveys shows that female circumcision continues to decline among Egypt’s youngest generation of girls at risk. A comparison of pathways through which this change may have come about shows the most robust evidence for a direct association with SES measures. We also find indirect evidence that there is substantial confounding of unobservable characteristics across areas as indirectly assessed through area-level fixed effects, suggesting an important role for community norms. All regression models that include either community cluster-level or sub-governorate area-level fixed effects show that unobservable differences across communities are statistically significant, and that, together, substantially alter the estimated effects of the other independent variables (the estimated coefficients were systematically different across models with fixed effects versus random effects as confirmed via a Hausman test, p-val < 0.000 [19]). Notably, maternal education as one SES measure becomes *more* strongly associated with daughter FGC risk once unobserved differences across communities are controlled for. Mother’s labor force participation and household wealth also predict daughter’s circumcision risk, and together with maternal education, suggest that pathways related to SES improvements can help to explain the rapid change FGC in Egypt. Furthermore, these SES-related drivers are independently salient above and beyond the influences of expected social norms.

These results for maternal education corroborate previous findings [5,17], but our additional exploration of maternal education for a subsample of girls suggests that maternal education and daughters’ FGC outcome may be causally linked. Additionally, controlling for unobservable area-specific time trends in the fixed effects specification shows that there is little residual confounding in the estimate of mother’s individual education related to the pace at which different areas may be changing, whether in terms of unobservable economic or social development. When we substitute area- and cohort-specific educational attainment for women for individual mother’s realized education (i.e. replacing her education with that of her peers), we find that increases in area-level women’s education is also associated with decreases in FGC. We argue that area-level women’s educational attainment is likely to be exogenously determined as a result of government policies aimed at increasing schooling since the 1960s [18]. As such, our constructed measure of women’s educational attainment is likely to reflect the educational opportunities prevailing during school-age years. Notably, the generation of women that experienced the largest increases in educational attainment is the same generation of mothers for whom the large decline in circumcision is observed among their daughters in more recent years.

Our results further elucidate findings from the existing literature about the role of social media in relation to FGC in Egypt. We corroborate results from Suzuki and Meekers (2008) that show that media exposure is related to a reduced risk of circumcision. However, our comparison of estimates between random effects and fixed effects specifications indicate that, when smaller geographic units are used as fixed effects instead of merely five regions, then media exposure is not a significant predictor of circumcision risk. This suggests that media messages about FGC may partially explain differences in circumcision rates across communities, but do not account for differences in circumcision risks within communities. Rather, within communities, there may be very little variation in exposure to such messages, particularly if messages are targeted at the community level and supplied uniformly to all households within the community. Analyses that do not control for unobserved differences across communities, but only differences at higher levels of aggregation at the regional level, may be comparing very different contexts.

Finally, we also find that the relationship between FGC risk and mother’s empowerment is fragile. As measured by two constructed empowerment indexes, women’s empowerment is a significant predictor of girls’ circumcision risk across communities, but not within. However, there may be measurement limitations that contribute to the lack of findings rather than suggesting a truly null effect. In particular, empowerment indicators included in the EDHS may not be capturing the locus of control where decision-making regarding FGC lies as this decision tends to be made by women in both the household and within larger community as part of distinct social networks. Factors that influence a mother’s power in these circles may be quite different than those that influence her decision-making leverage over household resources or her tolerance for domestic violence. Future work using a more targeted empowerment constructs and FGC-relevant measures may help to further test the empowerment pathway for explaining differences in circumcision risk. More broadly, understanding intrahousehold relations, social network influences, and the socio-cultural context in which mothers negotiate the circumcision status of their daughters well before daughters reach actual circumcision age requires a more mixed-methods approach for linking quantitative findings with qualitative depth. While a previous generation of socio-anthropological studies has done this in Egypt (e.g. [3,12,20]), a new round of studies is needed to understand the rapidly changing context in which circumcision preferences and practices are also changing, particularly with respect to gender roles, religion, and concepts of modernity.

While this study has leveraged nationally-representative samples to more completely compare different pathways hypothesized to explain the unprecedented decline of FGC in Egypt, our results should be interpreted in light of additional caveats. First, self-reported circumcision status by mothers may underreport circumcision given that the practice was becoming more and more stigmatized by the time of the 2005 and 2008 EDHS waves. Second, pervasive patriarchal views about women’s sexuality and marriageability would suggest a larger role for father’s education in daughter’s circumcision outcome. However, studies show that mothers are often the immediate decision-maker about the type and timing of a daughter’s circumcision (e.g., [17]) and that paternal education is not associated with the odds of circumcising daughters. Third, although comparing differences between random and fixed effects specifications provide suggestive evidence of the significant influence of unobservable heterogeneity across communities, we cannot further test for specific sources of heterogeneity. Fourth, even though the educational policy history in Egypt suggests our area- and cohort-specific measures of women’s educational attainment may be plausibly exogenous, more direct measures of supply-side schooling expansion (e.g. number of schools, female teachers, classrooms) are needed for rigorous testing of the education pathway and any causal relationship therein. Lastly, because of the restricted nature of the subsample used in the final analyses, these results may not fully generalizable particularly because they exclude all the Frontier governorates and mothers who moved.

## Conclusion

Overall our analyses suggest that women’s empowerment and social media appear to be more important in explaining differences across communities. Within communities, socioeconomic status indicators, and particularly mother’s education, are consistently and robustly related to girls’ circumcision outcomes. A series of additional analyses further suggest the relationship between women’s education and daughters’ FGC outcome may be causal.

## Endnotes

^a^While we recognize that “female circumcision” is a euphemism for the practice more commonly known as “female genital mutilation,” all national surveys conducted in Egypt use the term “circumcision,” or *khitan* in Arabic. This term is the same as the one used for male circumcision. Globally, the World Health Organization uses the terms “female genital mutilation” or “female genital cutting” (FGM/C). We choose to use the term circumcision throughout this paper to more accurately reflect the Arabic translation used in surveys conducted in Egypt. In this paper, “female circumcision” is synonymous with “female genital mutilation” and “female genital cutting.”

^b^The median age of circumcision is about nine years old and heavily skewed; of those who are circumcised, nearly 90 percent are circumcised by age 13.

^c^Including cluster fixed effects can account for any bias in estimates due to unobserved, time-invariant differences across communities because only within-community variation is used to produce regression coefficient estimates. Consequently, observations for which there is no within-community variation in the outcome, circumcision status, must be dropped from the analysis, reducing the sample size. By using a larger geographical unit, more variation in the outcome measure is available within area-level units and more observations can be retained in the regression model.

^d^We calculate the variance inflation factor, a metric used to quantify the severity of multicollinearity in regression analysis. We found that the variance inflation factor was lower than the cut-off suggested by statisticians above which multicollinearity begins to affect the parameter estimates in the analysis.

## Abbreviations

CI: Confidence interval; EDHSs: Egypt Demographic and Health Surveys; FE: Fixed effects; FGC: Female genital circumcision; OR: Odds ratio; PCA: Principal components analysis; RE: Random effects; SD: Standard deviation; SES: Socioeconomic status; SYPE: Survey of Young People Egypt; YOB: Year of birth.

## Competing interests

The authors declare they have no competing interests.

## Authors’ contributions

Authors contributed equally to this work. SM and JXL conceived of the idea. SM and JXL gathered the data. SM conducted the analysis. SM and JXL structured and wrote the manuscript. Both authors read and approved the final manuscript.

## Pre-publication history

The pre-publication history for this paper can be accessed here:

http://www.biomedcentral.com/1471-2458/13/921/prepub

## Supplementary Material

Additional file 1 Defining sub-governorate area units. Click here for file
