# Supplementary material for: Exploration of pathways related to the decline in female circumcision in Egypt
Source: BMC Public Health. 2013 Oct 3;13:921. doi: 10.1186/1471-2458-13-921 (PMC3835453; doi:10.1186/1471-2458-13-921)

**Additional file 1: Defining Sub-governorate Area Units**

To obtain a longitudinal measure of women’s educational attainment across birth cohorts, all waves of the EDHS can be used to leverage maximal number of observations. However, sampled clusters in the EDHS are randomly chosen and rarely are repeated across waves. Therefore, to obtain consistent geographical units for which educational attainment can be estimated from different waves, we match cluster locations in all subsequent survey waves to pre-defined sub-governorate units only available in the 1992 EDHS wave. Egypt is divided into 26 governorates, which are further divided into smaller administrative units—kism/shiakha in urban areas and markaz/villages in rural areas.

The 1992 EDHS sub-governorate area units are stratified across urban and rural areas and comprised of about three clusters on average, ranging from one to six. The average distance between two clusters in the 1992 wave is 4.7 kilometers for urban areas and 14.1 kilometers for rural areas. The GIS coordinates for each of the 1992 clusters within an area unit are triangulated to determine a central point, or centroid. Then, using the GIS coordinates of all clusters sampled in the 1995-2008 surveys, we find up to the 30 closest clusters to this centroid. To exclude more distant communities that may be less similar to others in the area, clusters more than 20 kilometers (12.4 miles) from the centroid in urban areas or more than 30 kilometers (18 miles) away in rural areas are dropped. Figure S1 illustrates this process. First, in the left panel, the GIS coordinates of four clusters coded to a given area unit in the 1992 EDHS are used to determine the center point of the area, from which a radius of 20 kilometers is drawn. In the right panel, the 24 closest sampled clusters in 1995-2008 EDHSs that fall within the centroid’s radius are coded to the area unit. The final sub-governorate unit mapping encompasses 98 percent of all urban clusters and 93 percent of all rural clusters in the 1992 EDHS. While not strictly based on administrative distinctions or precise neighborhoods, these GIS-based area units should reflect schooling opportunities both in terms of availability and norms in an area.

Using pooled data for ever-married women from all EDHSs (1992-2008), we then calculate the average educational attainment of all women within each area by cohort. Pooling across clusters allows for a more precise calculation for each birth cohort, spanning 25 years (1956-1980) within each geographic area. To ensure that each mother’s realized education does not drive variation in the constructed instrument for her area’s cohort, we exclude women who are mothers in our daughter circumcision outcomes dataset. We then create a five-year moving average of the proportion of women achieving at least a primary school level education within each area unit. Within any given year of birth and area cell, there is an average of 10 women (range 2-33), and smoothing over five years (two lagged years and two lead years) effectively provides a sample of about 50 women per area-cohort cell from which to calculate this figure. These measures are used in one final regression, Table 3 column 4 in the main text.

**Figure S1: Defining sub-governorate units.**


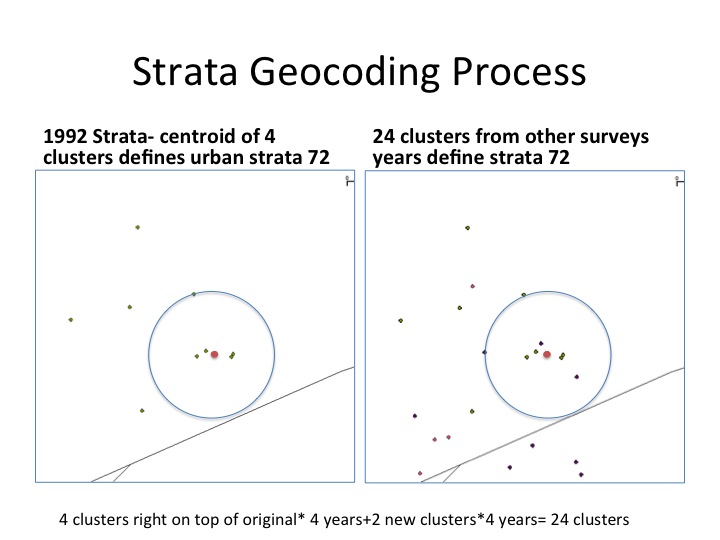

Supplement: Additional file 1 — Defining sub-governorate area units. [file 1471-2458-13-921-S1.docx]
